# Long-range and high-precision localization method for underwater bionic positioning system based on joint active–passive electrolocation

**DOI:** 10.1038/s41598-023-48957-x

**Published:** 2023-12-06

**Authors:** Meijiang Hou, Hailong Wu, Jiegang Peng, Ke Li

**Affiliations:** School of Automation Engineering, University of Electronic Science and Technology of China, Chengdu, 611731 China

**Keywords:** Physical oceanography, Biological physics

## Abstract

Active electrolocation organ of weakly electric fish act as a proximity detection system with high accuracy in recognizing object parameters such as size and shape. In contrast, some fish with passive electrolocation organ are able to detect objects at a greater range. This paper proposes a joint active–passive electrolocation algorithm for long-range and high-precision underwater localization, inspired by the active and passive electroreceptive organs of fish. The study begins by designing a large experimental platform for the underwater localization system to investigate the response of underwater objects to active and passive electric fields. Based on the response, the paper proposes separate underwater active and passive electrolocation algorithms, which are then combined to form a joint algorithm. Experimental results demonstrate that the proposed algorithm achieves high localization accuracy and long detection distance. The joint active–passive electrolocation algorithm has potential applications in submarine resource exploration, underwater robotics, and maritime military projects, while also providing new ideas for future research on long-range underwater object detection and identification based on electrolocation.

## Introduction

As society progresses and science and technology continue to develop, positioning technology is becoming increasingly sophisticated. Various forms of positioning technology, such as satellite positioning technology, base station positioning technology, and Wi-Fi positioning technology^1–4^, are widely applied in aviation, aerospace, navigation, surveying and mapping, military operations, natural disaster prevention, location search, vehicle navigation, personnel search and rescue, and numerous other fields. This has made it an indispensable and significant aspect of social life. However, although there are numerous positioning technologies available, most are only applicable to terrestrial or aerial environments. Given the complexity of the underwater environment, many of the existing positioning technologies are no longer suitable. As one of the important technologies for exploring underwater environment, underwater positioning technology has great application value in underwater resource exploration, underwater military construction and underwater robot navigation. In the ocean, the main technologies that can achieve positioning are acoustic, optical and GPS^5–7^. The advantages of acoustic methods are long positioning distance and mature technology. However, the acoustic method is easily affected by the environmental conditions of the water body, such as the thermocline in the ocean, bottom characteristics, tidal velocity, etc. The optical method can reach the centimeter level in close range positioning, but the method is greatly affected by the clarity in seawater. GPS has the advantages of high positioning accuracy, good flexibility and easy operation on land. However, its signal cannot be propagated in the underwater environment, and the realization of underwater positioning requires the use of other equipment, which makes the positioning cost increase^8^. Due to the limitation of these methods in underwater environment, we need to study other methods suitable for underwater positioning.

In fact, as early as the middle of the last century, biologists discovered a primitive sense for underwater localization, which came from a fish in nature, the weakly electric fish. Weakly electric fish can recognize their external environment in the dim, murky ocean in order to hunt for prey and avoid obstacles and natural predators because of a special ability they have, namely, electrolocation. Weakly electric fish have organs on their tails composed of special electric disclike cells called electric organ discharges (EOD), which can emit a voltage signal of a certain size to create an electric field around their bodies. Its body epidermis is distributed with electric field receptors, which are extremely sensitive, complex and practical, almost replacing the function of vision and smell in their daily work^9^. This means that understanding and imitating the localization mechanism of weak electric fish can provide us with a new underwater localization method.

There are two types of electrolocation, active and passive^10,11^. Active electrolocation is that the weakly electric fish establishes a low-frequency electric field around its body through the EOD and receives the electric field through the electric field receptors. When an object enters the electric field area established by the weakly electric fish, the electric field will change, and the changed electric field signal is received by the electric field receptor organ of the weakly electric fish and transmitted to the brain of the weakly electric fish for processing and analysis, so as to recognize the existence of the object and determine the location of the object, but the locating range is very limited^12^. This is because the active electric field emitter is like a pair of electric dipoles, and the intensity of the generated electric field decays with the cube of the distance^13^. Passive electrolocation are widely found in weakly electric fish, rays, sharks, catfish, and cetaceans, which sense electric field signals from the outside world through their electric field receptors and then identify and locate the object emitting the electric field signal^14–17^. In contrast to active electrolocation, passive electrolocation has a much wider effective range, and these fish are able to locate objects and communicate with other fish at distances greater than the active electrolocation distance^18^. Because the electric field received by passive electrolocation is generated by other organisms, the magnitude of this electric field is inversely proportional to the square of the distance^19^. Inspired by the above electric field perception of fish, many methods of underwater electric field detection have been proposed. For example, in active electric field detection, Bai et al. proposed an algorithm implemented in a robotic active sensing system that can recognize the size, shape, orientation and position of ellipsoidal objects with high localization accuracy, but the recognition range is only 20 cm^20^. Jiang et al. proposed an underwater moving object localization method based on the active electric field induction principle and long and short term memory network, and the experimental results show that the method has a two-dimensional average absolute localization error of 5.38 mm and a two-dimensional average relative localization error of 1.06% in a 500 mm $\times$ 80 mm area, which means that the localization error of the method is small and the localization accuracy is high^21^. In terms of passive electric field detection, Cho et al. proposed a real-time underwater object detection method using the geophysical direct current resistivity technique, and validated it in the field at sea. The method belongs to passive electric field detection, which can detect moving objects at a distance of 1 m away in real time, and the distortion response becomes clear when the excitation current increases to more than 20 A^22^. Jun-Zheng Zheng et al. proposed a localization scheme for a small underwater robot that swims freely in a large-scale environment^23^. The localization distance of this scheme is related to the designed transmitter, whose maximum detectable range is about 2.32–2.76 m, and the error has a large impact on the positioning accuracy. In summary, the active electric field can detect the size and shape of an object and can locate the position of the object, but the electric field strength decreases rapidly with distance, making the effective localization distance very limited. Compared with the active electric field, the passive electric field has a wider localization range, but its localization accuracy is lower.

Due to the complexity and uncertainty of the underwater environment, underwater electric field localization faces many challenges, such as changes in water temperature, salinity, and current velocity. To overcome these challenges, algorithms need to be designed to handle these complexities and improve the accuracy and reliability of underwater electric localization. Algorithms can extract useful information from the signal and reduce the influence of interfering signals to achieve accurate localization and tracking of underwater targets. Due to the relatively late development of underwater electric field localization technology, the research value of this technology is still to be explored, which makes that so far there are still only a few researchers engaged in this area of research, and even fewer research on underwater electric field localization algorithms, and most of the localization algorithms are designed according to the specific localization system in their research, and their universality is still to be further studied. For instance, in passive electric localization, Kim et al. proposed an axis calibration method for underwater electric field measurement sensors, and for the sensing array they designed, they used the Levenberg optimization algorithm to optimize the measured values, and the simulation results show that the algorithm can effectively reduce the localization error of their proposed axis calibration method for underwater robots^24^. In addition, Shang et al. proposed an underwater target localization method combining the subspace scanning algorithm and the meta-evolutionary programming particle swarm optimization algorithm, and they used this method to simulate and analyze the localization performance of uniform circular and cross-shaped electrode arrays, and the results showed that the method can effectively improve the localization accuracy without changing the localization accuracy and search speed^25^. In terms of active electric field localization, Yesol et al. proposed an algorithm that can decompose the measurement signal into multiple frequency coefficients and verified the feasibility of this algorithm in a localization system designed by them, in which the sensing arrays are multiple receiving electrodes arranged in a line^11^. Sylvain Lanneau et al. designed an algorithm that can be used for shape estimation and location localization of an elongated probe consisting of four metal rings and used a multi-signal classification algorithm to localize objects in a localization study^26^. Xu et al. proposed an active localization method based on a hybrid polarization multi-signal classification algorithm, and simulations and experimental analyses of a uniform circular antenna were performed to verify the effectiveness of the method^27^.

All of the above positioning methods are realized by designing different sensing arrays and using corresponding algorithms to locate the object or the positioning system itself. And our team mainly studies the controlled underwater electric field localization system, that is, by controlling the movement of the localization system, so that it is constantly close to the measured object, and when the movement of the localization system is within a certain range near the measured object, it indicates that the localization is successful. In terms of localization algorithms, our team has also conducted extensive research on algorithms applicable to controlled underwater electric field localization systems. In the preliminary research, our group found that the feature maps based on the time-domain data processing method would generate a lot of burrs due to the presence of environmental noise^28^, so the performance of the time-domain localization algorithm is easily affected by the environmental noise. In order to improve the anti-interference ability, robustness and stability of the underwater electric positioning system, our group designed a one-dimensional underwater frequency domain positioning algorithm based on FFT feature extraction^29^. On this basis, our team proposed a two-dimensional underwater active electrolocation system and studied three frequency domain localization algorithms, namely cross location algorithm, stochastic location algorithm and particle swarm optimization algorithm^30^. By comparison, the particle swarm optimization algorithm has the best overall performance. However, although the system based on particle swarm optimization algorithm can locate the underwater target, the algorithm is prone to fall into local optimum, which leads to the failure of localization. Therefore, we need to improve the algorithm or find other algorithms that are more suitable for controlled underwater electric field localization systems.

In this paper, we propose a novel approach to overcome the limitations of both active and passive electrolocation by combining their strengths. Our idea is based on the team’s extensive research on bionic systems and the development of a large experimental platform for the study of underwater electrolocation. The response characteristics of both active and passive electric fields have been thoroughly studied using the experimental platform, and a joint active–passive electrolocation algorithm has been designed based on these findings. The algorithm incorporates three nature-inspired optimization algorithms, namely particle swarm optimization (PSO), simulated annealing (SA), and hill climbing. Among them, PSO and SA form the PSOSA combined optimization algorithm, which improves on the separate PSO and SA, and is used for localization when the active electric field is operating in the combined active–passive electrolocation system. Whereas, the hill climbing algorithm is used for localization during passive electric field. The feasibility of the proposed algorithm has been verified through extensive experiments, and the results indicate that it can accurately position underwater targets over long distances. This combination of active and passive electrolocation provides a promising solution to address the challenges faced by traditional electrolocation methods.

## Research on the response characteristics of underwater electric field

**Figure 1:**
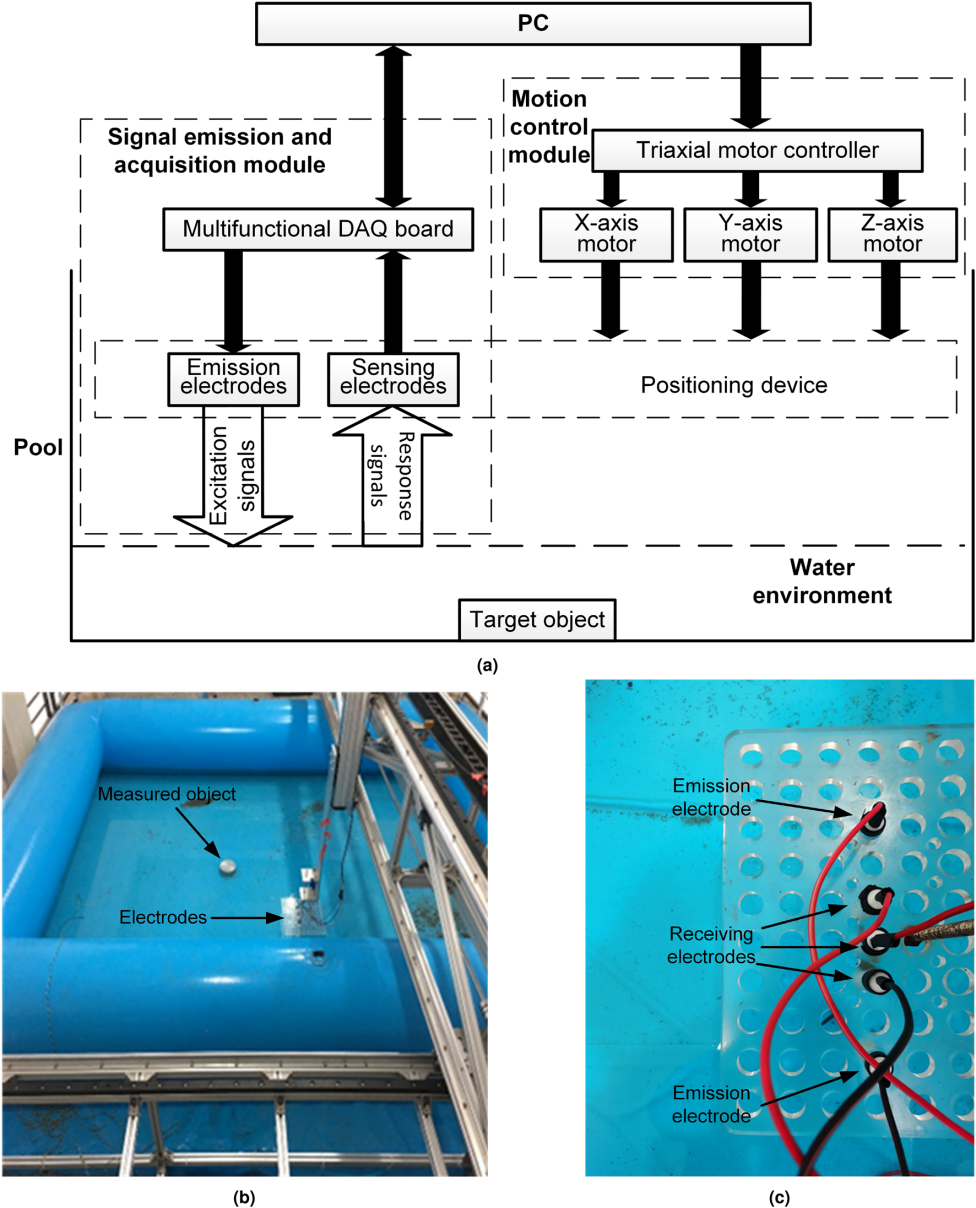
The experimental platform. (**a**) Schematic diagram; (**b**) physical diagram; (**c**) electrodes arrangement.

To achieve underwater electric field localization, we built an underwater electrolocation system. The schematic diagram of the experimental platform is shown in Fig. 1a, and the experimental platform of the system is shown in Fig. 1b. The system consists of four main modules: an electric field signal transmission and acquisition module, a signal data processing and analysis module, a motion control module, and a pool that simulates the underwater environment. The electric field signal transmission and acquisition module is responsible for generating and transmitting an electric field, as well as acquiring the electric field signals reflected from the target object. The signal data processing and analysis module is responsible for processing and analyzing the acquired electric field signals to determine the location of the target object. The motion control module is responsible for controlling the movement of the underwater vehicle to the target object. The pool is used to simulate the underwater environment of the test system, and its size is 4 m $\times$ 2.9 m $\times$ 0.5 m. Figure 1c shows the arrangement of the electrodes, ordered from top to bottom, with the first and fifth electrodes being the active electric field transmitting electrodes; the second and fourth electrodes being the active electric field receiving electrodes; the third electrode being the passive electric field receiving electrode; and the other passive electric field receiving electrode being placed at the distal end of the pool and grounded. Noise in the experiments was suppressed by filters. All the following experiments were performed on this platform. In the research process, LabVIEW and Python were mainly used, where LabVIEW was used for the development of the experimental software and Python was used for the simulation of the algorithm.

**Figure 2:**
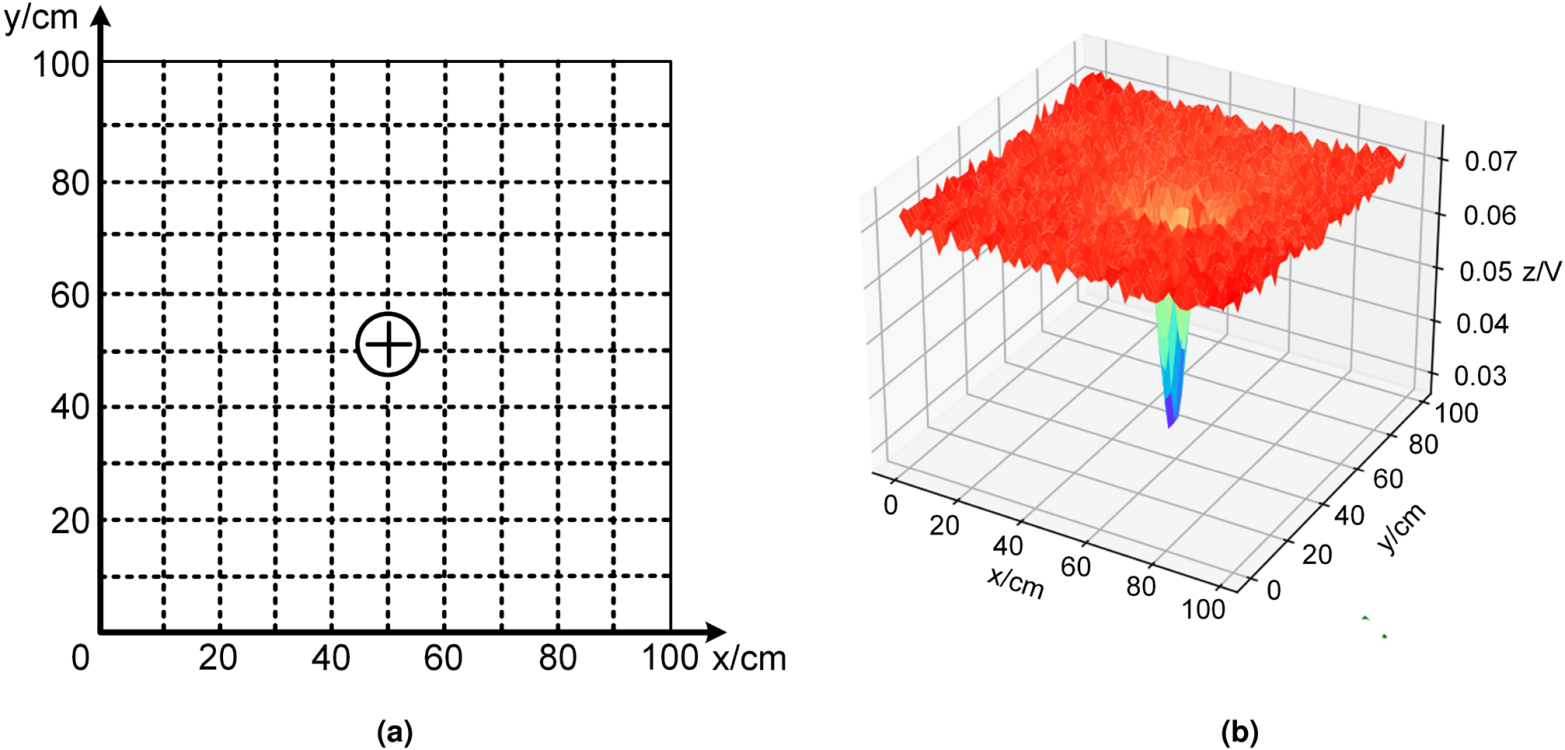
Response of active electric field. (**a**) The top view of the experiment platform; (**b**) the three-dimensional image of the response characteristics of the target object in active electric field.

As mentioned earlier, electric fish can sense the presence of objects in the water using low frequency electric fields. When an object enters the electric field environment established by the electric fish, it distorts the electric field and produces a corresponding change in voltage amplitude, which is then sensed by the electric fish. In addition, the electric fish can determine the direction and position of the object and localize it by detecting the weak electrical signals emitted by the object. In order to study the response characteristics of the object under test to active and passive electric fields, we placed a solid aluminum cylinder as a target object in a pool and measured the voltage amplitude around it by controlling the motion of the underwater electric positioning system. Figure 2 shows the response test of the active electric field with the target object placed at the coordinates x = 50, y = 52 of a 100 cm by 100 cm grid covering the exploration area. Figure 2a illustrates the location of the target object, while Fig. 2b shows the characteristics of the response of the target object to the active electric field, where the projection of the lowest point of the amplitude in the xy-plane indicates the location of the target object. From the figure, it can be seen that when the localization system is far away from the target object, the characteristic values of the collected electric field response float around the amplitude of 0.072 V, and the absolute value of the fluctuation range is within 0.01 V. When the localization system is within 5 cm of the target object, the response value decreases sharply, and the minimum response value of 0.027 V occurs in the location of the target object.

**Figure 3:**
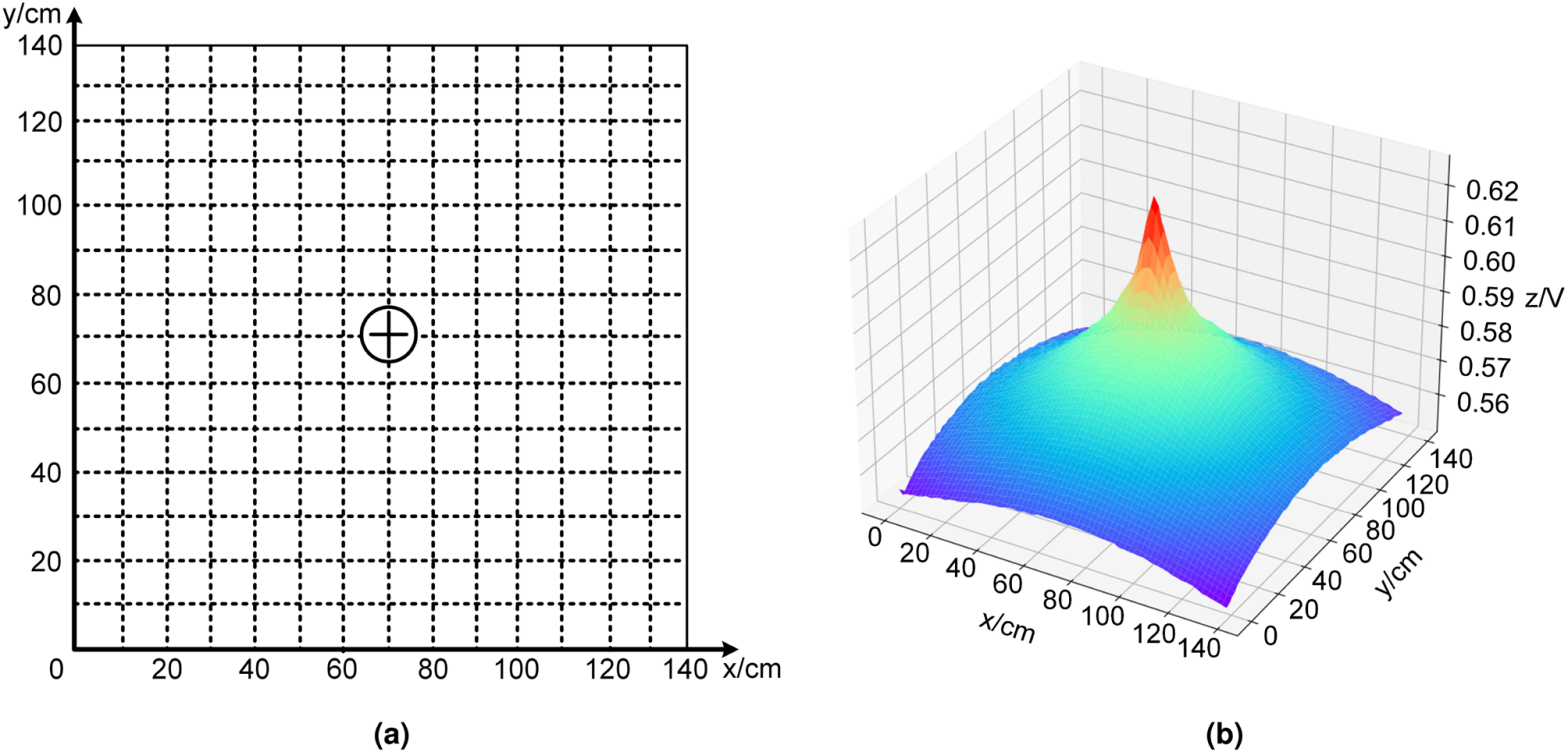
Response of passive electric field. (**a**) The top view of the experiment platform. (**b**) The three-dimensional image of the response characteristics of the target object in passive electric field.

In the test of the response of the target object to the passive electric field (Fig. 3), the target object was placed at the coordinates x = 70, y = 70 of a 140-cm by 140-cm grid covering the exploration area, and a signal source was placed at its bottom to simulate that it could emit an electric signal, as shown in Fig. 3a. Figure 3b represents the response characteristics of the target object to the passive electric field, where the projection of the highest point of amplitude in the xy-plane indicates the location of the object. From the figure, it can be seen that as the detection distance of the localization system decreases, the characteristic value of the collected electric field response gradually increases. Especially when the positioning system is far away from the target object, the slope of the eigenvalue change is small, but as the distance decreases, the slope of the eigenvalue change becomes larger and larger until it reaches the maximum value at the target object.

The experimental results reveal that the response characteristics of the active electric field exhibit rapid fluctuations in proximity to the object, and exhibit minimal variations at a distance, which hinders its utility for long-range positioning. In contrast, the response characteristics of the passive electric field exhibit a desirable curve, with a clear gradient of the electric field that varies with distance, making it favorable for long-range positioning. Subsequently, we shall investigate localization algorithms that are based on the distinct response characteristics of the electric field.

## Localization algorithm design

From the previous section, it can be seen that there are significant differences in the response of the object under test to the active and passive electric fields. Therefore, in this section, the corresponding localization algorithms will be designed based on the characteristics of the object’s response to active and passive electric fields obtained in the previous section to realize active and passive electrolocation, respectively. Then, combining the active electric field’s ability to recognize more information about the object and the passive electric field’s ability to increase the detection distance, the two algorithms are combined to form a joint active–passive electrolocation algorithm.

### Active electrolocation strategy based on bionic algorithm

In accordance with the response properties of underwater target objects within an active electric field, the precise localization of such objects can be achieved by identifying the nadir of the underwater electric field response value. This enables the transformation of the active electric field localization quandary into an optimization problem. An optimization problem, by definition, involves determining the maximum or minimum value of a particular objective function under specified constraints. Bionic algorithms, as heuristic methodologies derived from natural phenomena and biological entities, closely emulate natural behaviors. Since the design of the underwater active electric field system is inspired by biological organs, the use of bionic algorithms in active electric localization is a reasonable and effective choice. Through the bionic algorithm, the behaviors and mechanisms of living organisms can be simulated, which makes the positioning system more in line with natural behaviors and improves the accuracy and adaptability of positioning. In addition, since the expression of the objective function in the localization process is unknown, traditional optimization algorithms may not be directly applied. Therefore, the use of meta-heuristic bionic algorithms is a successful way to achieve localization. The meta-heuristic bionic algorithm is able to simulate the behavior of organisms in nature and gradually approach the optimal solution by searching and optimizing, so as to achieve the localization goal in the case of unknown objective function.

At present, the common meta-heuristic algorithms include simulated annealing algorithm (SA), genetic algorithm (GA) and particle swarm optimization algorithm (PSO)^25,31,32^. The SA is inspired by the behavior of solid matter in bionics as it changes with temperature. In optimization problems, the SA searches for the global optimal solution by the degree of random variation of states in the temperature control system. The basic idea is to introduce a certain degree of randomness in the search process in order to overcome the distress caused by the multi-peaked problem, so that the algorithm has enough chances to jump out of the local minima and find the global optimal solution. SA has the advantages of being simple and easy to implement, and having a strong ability of global search, however, the algorithm is slow to converge and requires a large amount of computational resources.GA are inspired by the evolutionary theory in bionics. In optimization problems, GA is an optimization algorithm that simulates the mechanism of natural selection and genetic inheritance, and gradually optimizes the population by three basic operations: selection, crossover and mutation, so that the population gradually converges to the global optimal solution. Its greatest advantage is its parallelism, which is suitable for solving optimization problems with high dimensionality, but its convergence speed is slow. PSO is inspired by the collective behavior of flocks of birds or schools of fish in bionics. In the optimization problem, the PSO achieves efficient solution of the optimization problem by simulating the process of “individual” following and “group” learning and collaboration of particles. The basic idea is to treat each individual as a particle, and use the historical optimal position and the global optimal position as a guide to continuously adjust its own position and speed, so that the algorithm gradually approximates the global optimal solution. Its advantages are simple and easy to implement, better adaptability to the constraints of the problem, and parallelizable processing, however, the algorithm is sensitive to the local optimal solution of the problem, and it is easy to fall into the local optimal solution.

Its advantages are simple and easy to implement, better adaptability to the constraints of the problem, and parallelizable processing, however, the algorithm is sensitive to the local optimal solution of the problem, and it is easy to fall into the local optimal solution. The response characteristic value of underwater active electric field is taken as the model of optimization problem. In the simulation experiment, 0.027 V, minimum value, is used as the criterion to judge whether the positioning is successful. Three algorithms, PSO, GA and SA, are simulated for 1000 times respectively. The success rate of the three algorithms in 1000 simulation tests, as well as the fastest, slowest and average iterations when successful are analyzed and explored. The simulation results are shown in Table 1.

**Table 1:** Simulation performance comparison of three algorithms.

Algorithm	Success rate (%)	Fastest iterations	Slowest iterations	Average iterations
PSO	97.8	1	1000	27.525
GA	76.5	2	1000	339.553
SA	100.0	2	817	125.904

From Table 1, it can be seen that the optimization success rate is 100% for SA, 97.8% for PSO and 76.5% for GA in 1000 tests. This means that SA succeeded in finding the optimal solution every time in conducting 1000 tests, whereas PSO and GA did not reach the optimal solution in a part of the tests, but the success rate of PSO is also relatively high. In terms of average number of iterations, the average number of iterations for SA is 125.904, PSO is 27.525 and GA is 339.553. This indicates that PSO’s algorithm is usually faster than SA and GA in finding the optimal solution, while GA requires the most iterations. Based on the data in Table 1, it can be seen that PSO performs best in terms of the number of iterations and also performs well in terms of the success rate; SA performs best in terms of the success rate but has a higher number of iterations; and GA performs the worst in terms of the success rate and the number of iterations, which is not applicable to our proposed localization system. Taken together, the above analysis allows us to explore a localization algorithm that is more suitable for active electrical localization. Combining the success rate advantage of SA with the speed advantage of PSO, the Metropolis acceptance probability criterion of SA is introduced into PSO, so as to improve PSO, and the improved algorithm is called PSOSA combined optimization algorithm.

**Figure 4:**
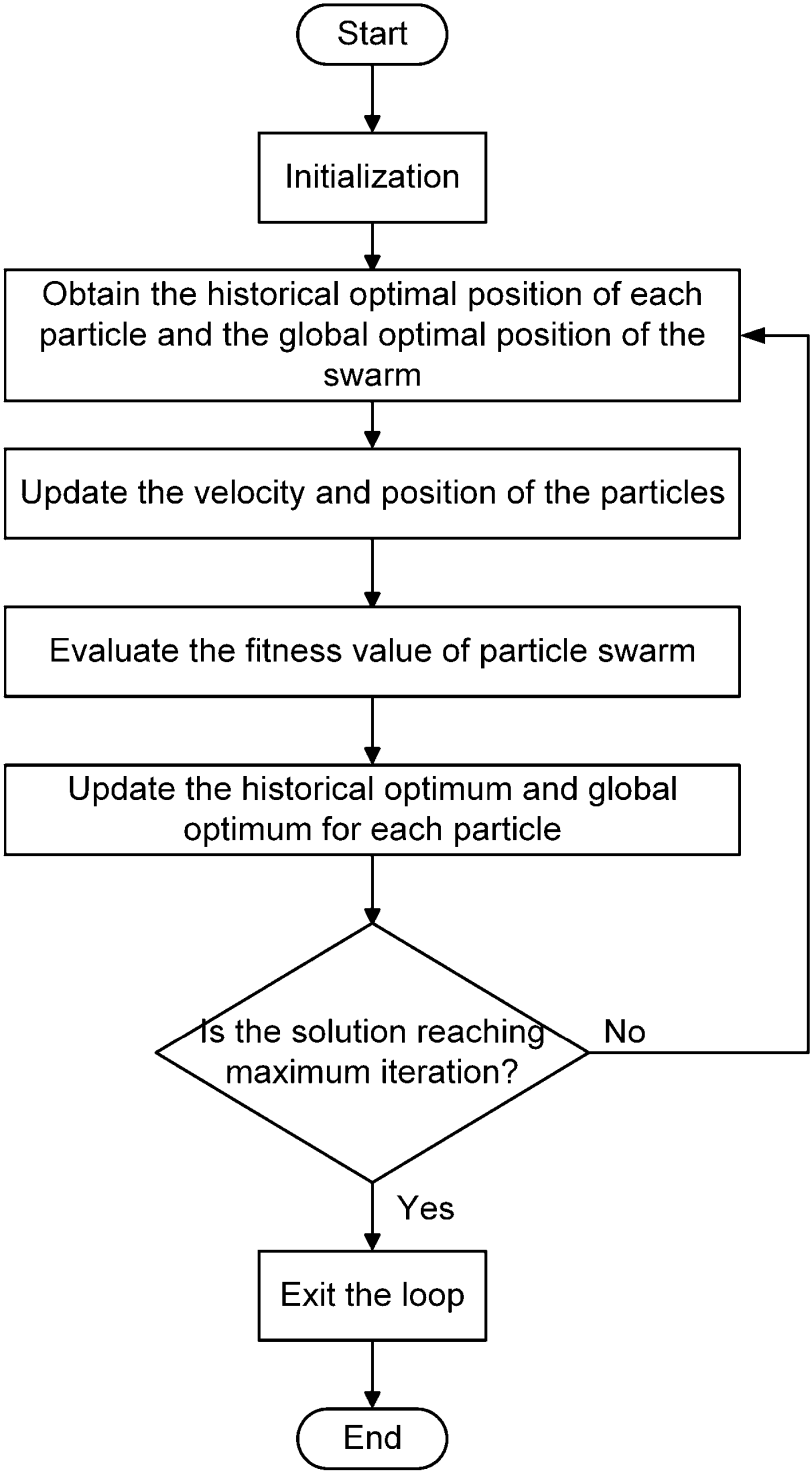
The process of PSOSA combined optimization algorithm.

In PSO, each particle will accept the new position calculated according to the position update formula with a 100% probability. In PSOSA combined optimization algorithm, Metropolis criterion will determine the probability of accepting new position according to the difference between the fitness values of the new position and the old position of the particle. This optimization makes it easier for the particle swarm to jump out of the local optimal solution in the update process, and the fluctuation of the overall fitness value will be reduced when the search approaches the end. Selecting the appropriate cooling method in the optimized algorithm determines the quality of the algorithm in the optimization process. Generally, when the search starts, the particle is in the highest temperature state, and will accept the bad new state with a great probability, so it is easy to jump out of the local optimal solution. When the temperature gradually decreases to the final stable state, the probability of the particle accepting the bad state is very low, so the particle can finally be in a relatively stable state. The cooling method selected in the PSOSA combined optimization algorithm is shown in Eq. (1):

1 $$\begin{aligned} {T_k} = \frac{{{T_{\max }}}}{{{k_0} + k}} \end{aligned}$$

where $T_{\max }$ is mean the initial maximum temperature; $T_k$ is the temperature of the kth iteration; $k_0$ is a constant. From Eq. (1), it can be seen that the temperature decreases slowly with the increase of iterations, and the temperature drops very quickly at the beginning of iteration, but very slowly at the end. This makes the algorithm update more at low temperature. Figure 4 shows the flow of the PSOSA combined optimization algorithm. The basic steps of PSOSA combined optimization algorithm are as below:

- Randomly generate the velocity and position of *n* particles in 2D search space.
- According to the underwater electric field characteristic response value at the position of each particle, the fitness value of each particle is evaluated, and the historical optimal position of each particle and the global optimal position of the swarm are obtained according to the fitness value.
- Update the velocity and position of particles according to the velocity update Eq. (2) and the position update Eq. (3)^33,34^:
  2 $$\begin{aligned} v_{id}^k = \omega v_{id}^{k - 1} + {c_1}\xi (p_{id}^{k - 1} - x_{id}^{k - 1}) + {c_2}\eta (p_{gd}^{k - 1} - x_{id}^{k - 1}) \end{aligned}$$

3 $$\begin{aligned} x_{id}^k = x_{id}^{k - 1} + v_{id}^k \end{aligned}$$

where subscript *d* is the dimension of search space; *k* is the iteration of the particle; $x_i$ and $v_i$ are the position and velocity of particle $i (i = 1, 2, 3, \ldots , n)$ respectively; $p_i$ is the optimal position of particle *i*; $p_g$ is the optimal position in the particle swarm; $\omega$ is the inertia weight; $c_1$ and $c_2$ are two acceleration constants of particle; $\xi$ and $\eta$ are two random numbers in [0, 1].

(d) Evaluate the fitness value of particle swarm.
(e) Calculate the fitness value difference between the new position and the old position of each particle: $\Delta f = {f_{new}} - {f_{old}}$.If $\Delta f < 0$ ,the particle will unconditionally accept the new position. Otherwise, particle will accept the new position according to Metropolis criterion, that is, the probability of particle accepting the new position in the algorithm is shown in Eq. (4):

4 $$\begin{aligned} P = P(old - new) = \left\{ \begin{array}{l} 1,\quad \Delta f < 0\\ \exp \left( { - \frac{{\Delta f}}{{{T_k}}}} \right) \end{array} \right. ,\quad \Delta f \ge 0 \end{aligned}$$

(f) Stop if the solution is good enough or reaching the maximum iteration. Otherwise, reduce the temperature according to the cooling method, update the weight value and go back to step (b).

In the iterative process of the algorithm, if the updated velocity $v_{id}^k$ exceeds the specified particle velocity range $\left[ {{v_{\min }},{v_{\max }}} \right]$, the boundary value is taken. If the updated particle position $x_{id}^k$ exceeds the search range $\left[ {{x_{\min }},{x_{\max }}} \right]$, the boundary value is also taken.

In the search process, inertia weight $\omega$ is an important factor to determine the ability of local search and global search, which is updated according to the linear adjustment strategy proposed by Shi and Eberhart as shown in Eq. (5)^34^:

5 $$\begin{aligned} \omega = {\omega _{\max }} - k\left( {{\omega _{\max }} - {\omega _{\min }}} \right) /{k_{\max }} \end{aligned}$$

where $k_{\max }$ is the maximum iteration; $\omega _{\max }$ and $\omega _{\max }$ are the maximum and minimum values of $\omega$, respectively.

In order to fully demonstrate the feasibility and effectiveness of the PSOSA combined optimization algorithm in the process of underwater target positioning, 1000 repeated simulation experiments were carried out for the PSO, SA and PSOSA combined optimization algorithms, and the results are shown in Table 2.

**Table 2:** Simulation result.

Algorithm	Success rate (%)	Average iterations
PSO	97.4	31.562
SA	100	133.741
PSOSA combined optimization algorithm	99.7	12.393

It can be seen from the above data, compared with PSO, the PSOSA combination optimization algorithm has obviously improved its success rate and speed. Although its success rate has not reached 100% success rate of SA, it can also reach a high success rate of 99.7%, and the average iterations is reduced to 12.393 in terms of speed. Therefore, PSOSA combination optimization algorithm not only improves the success rate, but also overcomes the disadvantage of slow speed of SA in the optimization process. Based on the above analysis, the PSOSA combinatorial optimization algorithm is selected as the active electrolocation method in this study.

### Passive electrolocation strategy based on bionic algorithm

As previously delineated in this manuscript, the hill climbing algorithm draws its inspiration from natural phenomena. This hill climbing algorithm can be characterized as an optimization technique grounded in the observed behavior patterns of living organisms, analogous to the actions of certain animals or humans as they ascend hills or mountains in pursuit of elevated positions. Within the context of the hill climbing algorithm, the solution to a given problem is conceptualized as the apex of a mountain, with the optimization objective being the identification of the highest point on said peak. The algorithm commences with the existing solution, examining the adjacent points within the solution space, and subsequently contrasting the optimization goal values of these points with the present point. Upon the discovery of a higher point, the algorithm shifts to that position and resumes the examination of neighboring points. This iterative process persists until the algorithm reaches a local summit and is incapable of locating a higher point.

The basic steps of hill climbing algorithm are as below:

- Randomly generate a starting position in 2D search space, and initialize the moving step size.
- Evaluate the fitness value of each position according to the underwater electric field characteristic response value.
- Compare the fitness values of the current position and four positions around the current position, which are one step size away from the current position. Select the position of the maximum value as the starting position of the next iteration.
- If the fitness value of the current position is greater than that of the four surrounding positions, the optimal position is found and the iteration is stopped. Otherwise, update the step size and go back to step (c).

**Figure 5:**
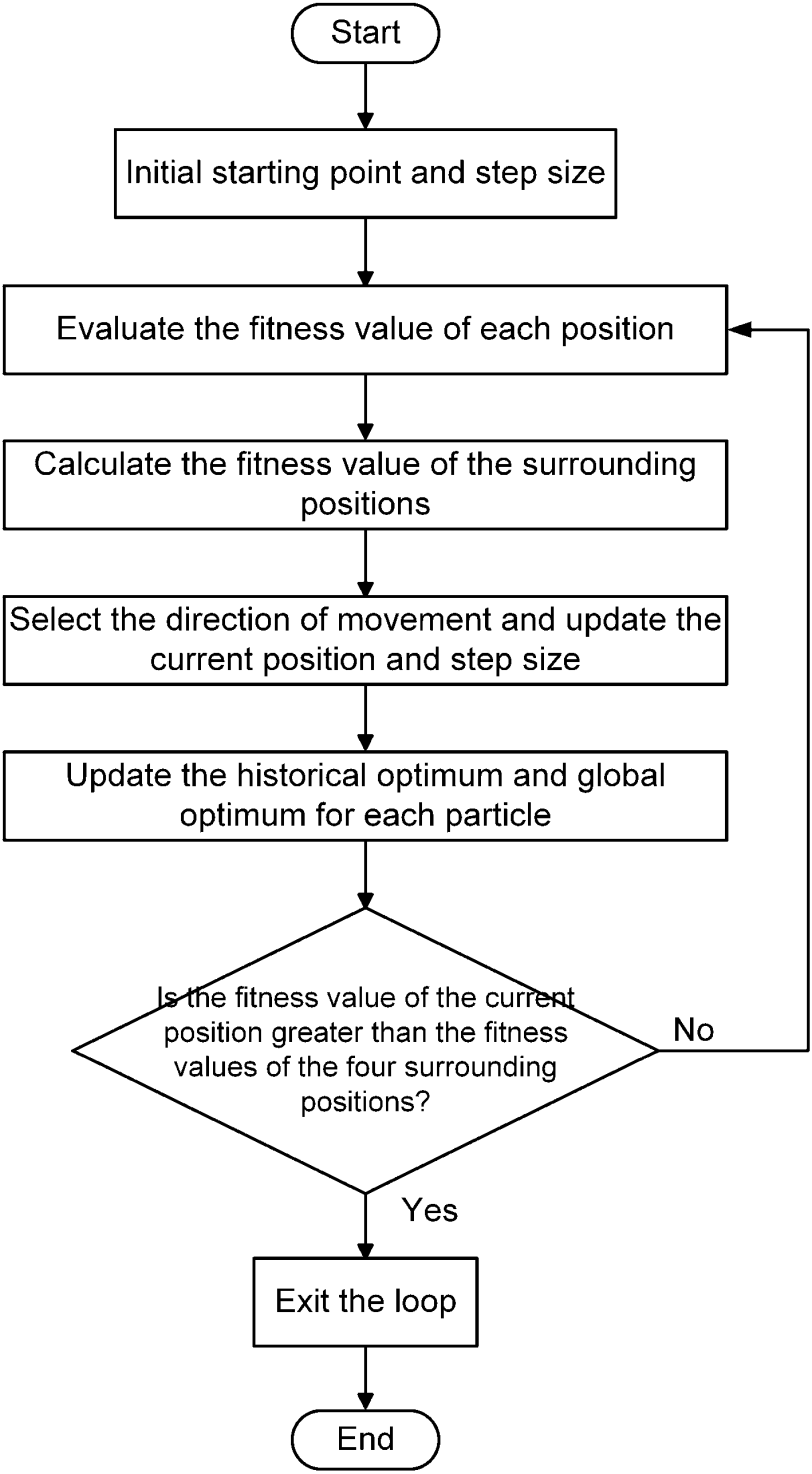
The process of hill climbing algorithm.

The flow of the hill climbing algorithm is shown in Fig. 5.

To prove the feasibility of hill climbing algorithm, we carried out simulation experiment on the hill climbing algorithm, taking the response characteristic value of passive electric field as the simulation model. In the simulation, the starting position is random, and the positioning is regarded as successful when the algorithm searches the highest point.After 1000 simulation experiments, we obtained the following results (see Table 3): the hill-climbing algorithm can successfully localize to the highest point from any position. Therefore, the hill-climbing algorithm can be applied to the underwater passive electric field localization problem.

**Table 3:** Simulation result of hill climbing algorithm.

Algorithm	Success rate (%)	Average iterations
Hill climbing algorithm	100.0	6.759

### Joint active–passive electrolocation algorithm

As stated in “Introduction” , the position, shape and size of an object can be identified using active electric field, but its detection range is narrow, which makes the active electrolocation limited to a small range of localization, while the passive electric field has a wider detection range, in order to make full use of the characteristics of the active and passive electric fields, we propose a new joint active–passive electrolocation algorithm. The basic idea of this algorithm is to first use passive electrolocation to guide the positioning device to the position near the underwater target, and then use active electrolocation to localize the target. Through passive electric positioning, we can determine the approximate position range of the target, and then use active electric positioning to further pinpoint the target. This joint positioning algorithm can make full use of the advantages of the two positioning methods to improve the accuracy and reliability of positioning.

**Figure 6:**
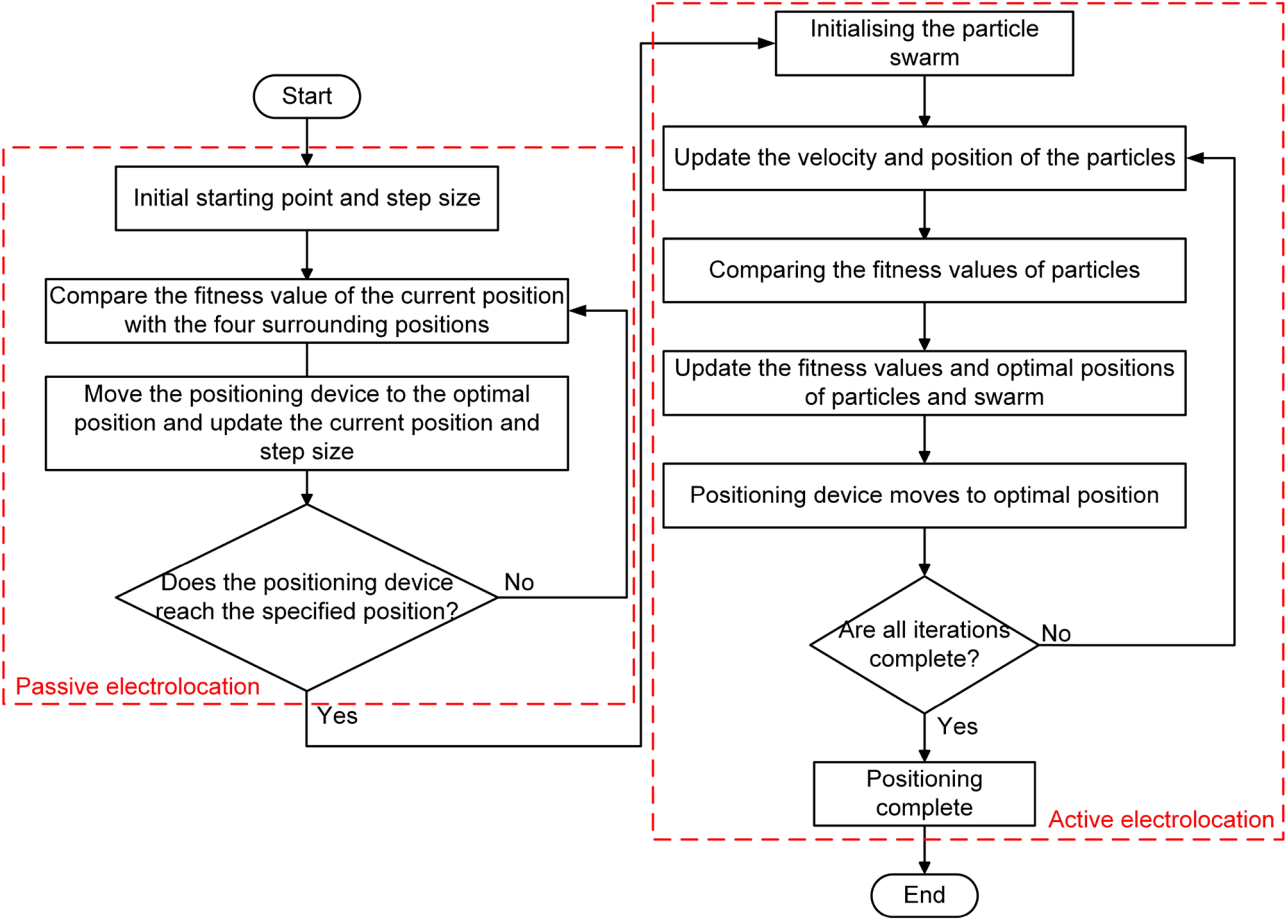
Schematic diagram of the effective positioning range and accuracy calibration of the two-dimensional plane.

The basic steps of the algorithm are as below:

- Use passive electrolocation, set the range centered on the target. Randomly generate a starting position in 2D search space, and initialize the moving step size.
- Compare the fitness values of the current position and four positions around the current position, which are one step size away from the current position. Move the positioning device to the position of the maximum value.
- If current position is within the set range, switch to active electrolocation, initialize the particle swarm. Otherwise, update the step size and go back to step (b).
- Update the velocity and position of particles. Compare the fitness value of particles, and then update the optimal fitness value and optimal position of particles and swarm. Move the positioning device to the optimal position of swarm.
- Stop if the solution is good enough or reaching the maximum iteration. Otherwise, go back to step (d). The process of joint active–passive electrolocation algorithm is shown in Fig. 6.

By combining passive and active electrical localization, we can search for a target over a wider range and acquire more information for more accurate localization.

## Verification experiment

**Figure 7:**
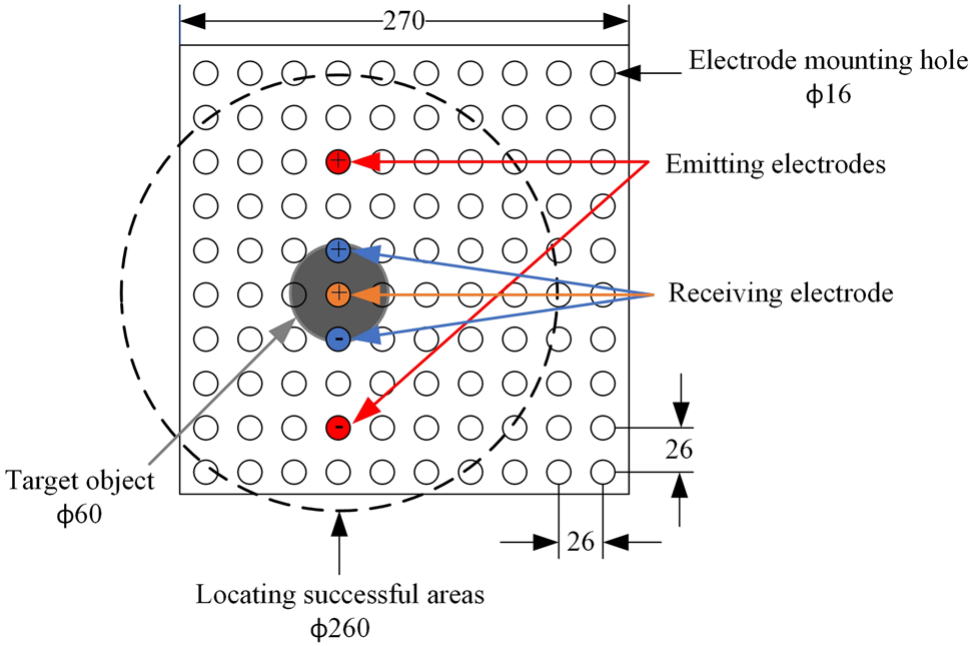
The process of joint active–passive electrolocation algorithm.

To validate the feasibility of the designed hill climbing algorithm, PSOSA combined optimization algorithm and joint active–passive electrolocation algorithm, we applied them to our designed underwater electrolocation system for underwater electrolocation experiments, respectively. In the experiment, both the transmitting and receiving electrodes are mounted on a bracket with a length of 270 mm $\times$ 270 mm, as shown in Fig. 7. The holder is evenly distributed with 100 holes of 16 mm diameter and used as a reference scale for positioning accuracy. The positioning accuracy in the experiment is expressed as the magnitude of the minimum distance between the nearest electrode to the edge of the target object and the edge of that object, and it indicates that the positioning system successfully locates the target object when the positioning accuracy is less than 100 mm. The target object used for the experiments in this section is an aluminum cylinder with a diameter of 60 mm, as shown in the gray circular surface in Fig. 7. In addition, the black dashed line in Fig. 7 indicates the successful localization range of 260 mm in diameter, i.e., when the object is located within the circle of the black dashed line, it indicates that our localization system has successfully localized the object.

**Figure 8:**
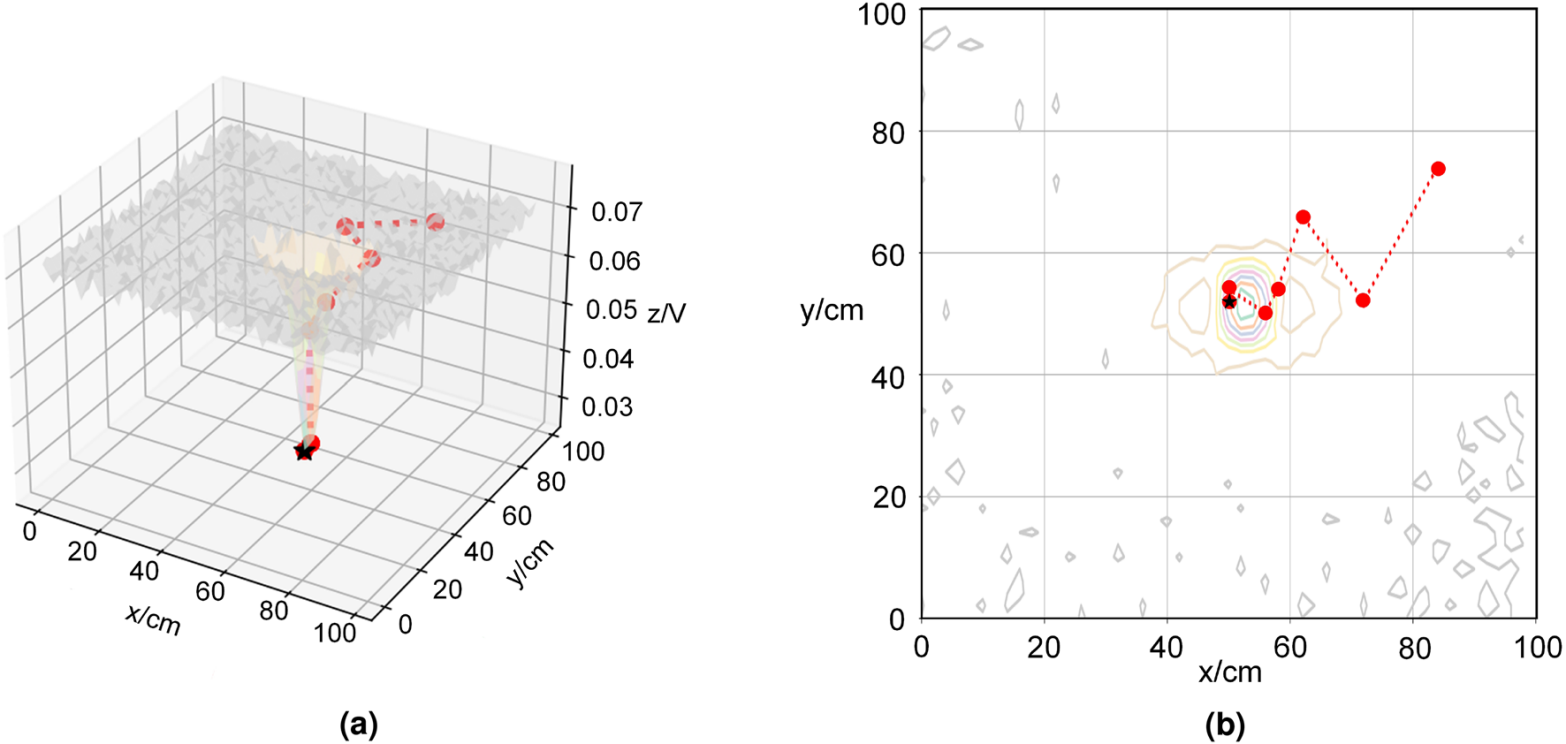
Result of active electrolocation. (**a**) 3-D; (**b**) 2-D.

In the experiments with the PSOSA combined optimization algorithm, the initial position of the positioning device is also used as the coordinate origin of the 2D detection plane. The target object is an aluminum cylinder placed at the center of the 100 cm $\times$ 100 cm 2D search space. The active electric field was generated by a continuous sinusoidal signal with an amplitude of 2 V and a frequency of 100 Hz. During the experiment, the positioning device is guided by the PSOSA combined optimization algorithm and moves automatically from the initial position step by step according to the detected values until it approaches the target position. The experimental results are shown in Fig. 8, in which the ring lines of different colors indicate the voltage amplitude contours around the target object, the red dots indicate the optimization position after each iteration of the algorithm, that is, the position of the detection device in the two-dimensional detection plane after each iteration of the algorithm, the red dashed line, which indicates the optimization path of the algorithm for each iteration, i.e., the motion path of the detection device with the algorithm iteration, and the black five-pointed star is the actual position of the target object. The black pentagram is the actual position of the target object. Figure 8a shows the optimization process of the PSOSA combined optimization algorithm. In this experiment, the response characteristics of the target object to the active electric field are similar to Fig. 2b. From the figure, it can be seen that the detection device gradually approaches the target object as the number of iterations increases, and reaches the location of the target object after six iterations. Figure 8b shows the motion trajectory of the localization device on the 2D detection plane. The experimental results show that the PSOSA combined optimization algorithm can locate the target object quickly and accurately.

**Figure 9:**
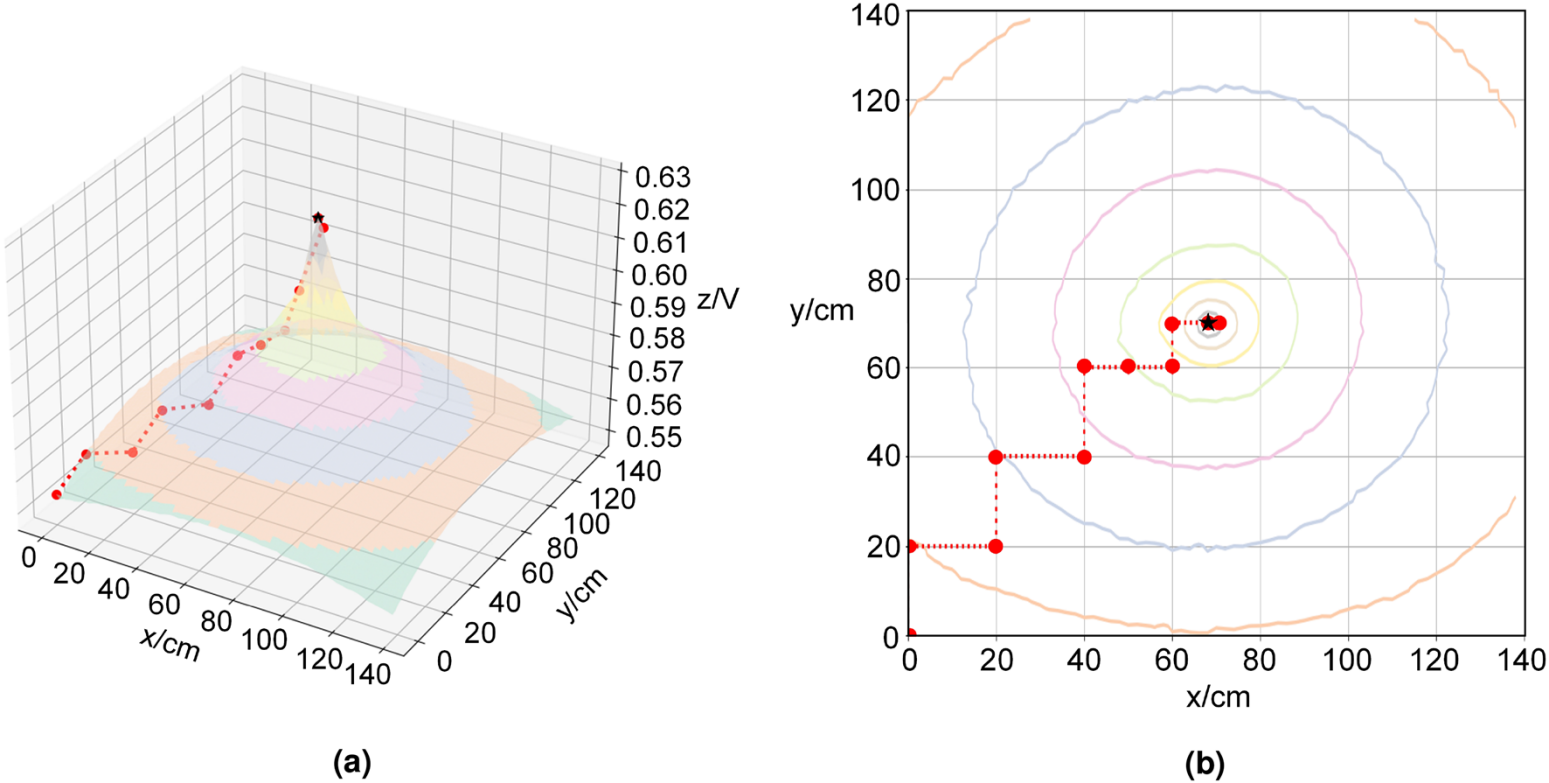
Result of passive electrolocation. (**a**) 3-D; (**b**) 2-D.

In the localization experiments using the hill climbing algorithm, the initial position of the localization device was used as the coordinate origin of the two-dimensional detection plane. The target object was located at the center of the 140 cm $\times$ 140 cm two-dimensional search space. A continuous sinusoidal signal with an amplitude of 2 V and a frequency of 100 Hz was emitted from the bottom of the target object. Similarly, during the experiment, the localization device was guided by the hill climbing algorithm to gradually move from the initial position until it approached the target position. The experimental results are shown in Fig. 9, in which the different colored ring lines indicate the voltage amplitude contours around the target object, the red dots indicate the optimal position of the algorithm after each iteration, i.e., the position of the positioning device around the target object, the red dashed line indicates the optimal path of the algorithm after each iteration, i.e., the position of the positioning device in the two-dimensional detection plane after each iteration of the algorithm, and the black five-pointed star is the actual position of the target object. Figure 9a shows the optimization process of the hill-climbing algorithm. In this experiment, the response characteristics of the target object to the passive electric field are similar to Fig. 3b. From the figure, it can be seen that the detection device gradually approaches the target object as the number of iterations increases. After 10 iterations, the location of the target object is reached. Figure 9b shows the motion trajectory of the localization device on the 2D detection plane. The experimental results show that the hill climbing algorithm can also locate the target object quickly and accurately.

**Figure 10:**
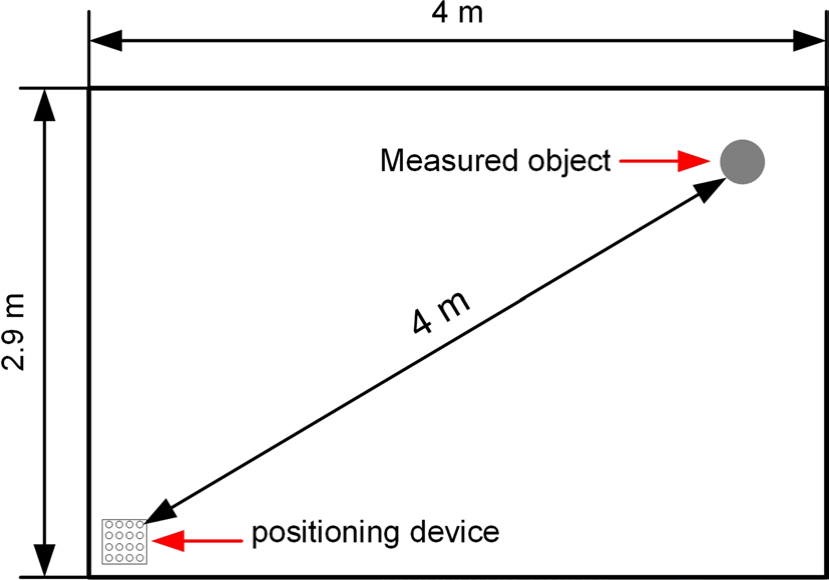
Schematic diagram of the joint active–passive electrolocation experiment in two dimensions.

In the experiments of the joint active–passive electrolocation algorithm, to highlight the localization performance of the algorithm, the initial position of the localization device was set at a distance of 4 m from the target object. Considering that the size of the pool is 4 m $\times$ 2.9 m $\times$ 0.5 m, in order to achieve the detection distance of 4 m, the localization device and the target object were respectively placed at the diagonal in the pool, as shown in Fig. 10. In this experiment, when performing passive electrolocation, the underwater electric field is provided by a signal source placed near the object with an amplitude of 15 V and a frequency of 100 Hz. After switching to active electrolocation, the transmitting electrode emits a sinusoidal excitation signal with an amplitude of 10 V and a frequency of 100 Hz.

**Figure 11:**
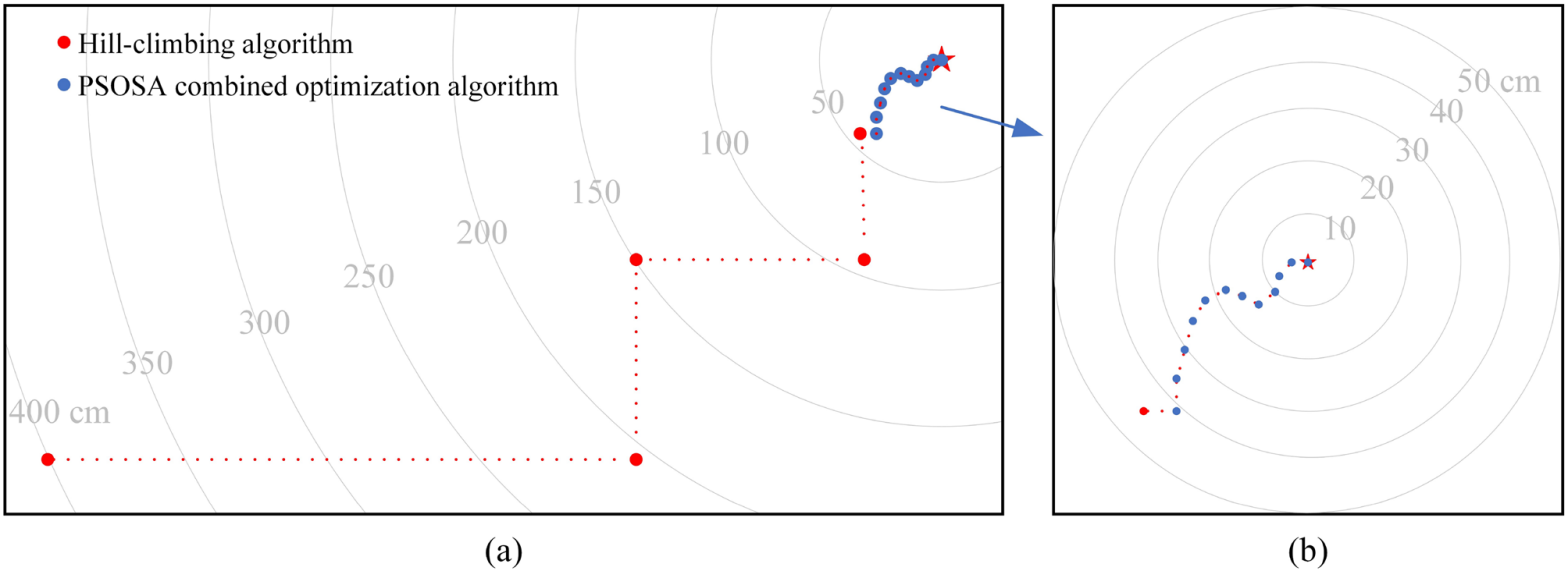
Schematic diagram of the localization path for joint active–passive electrolocation. (**a**) Joint active–passive localization path; (**b**) active localization path.

**Figure 12:**
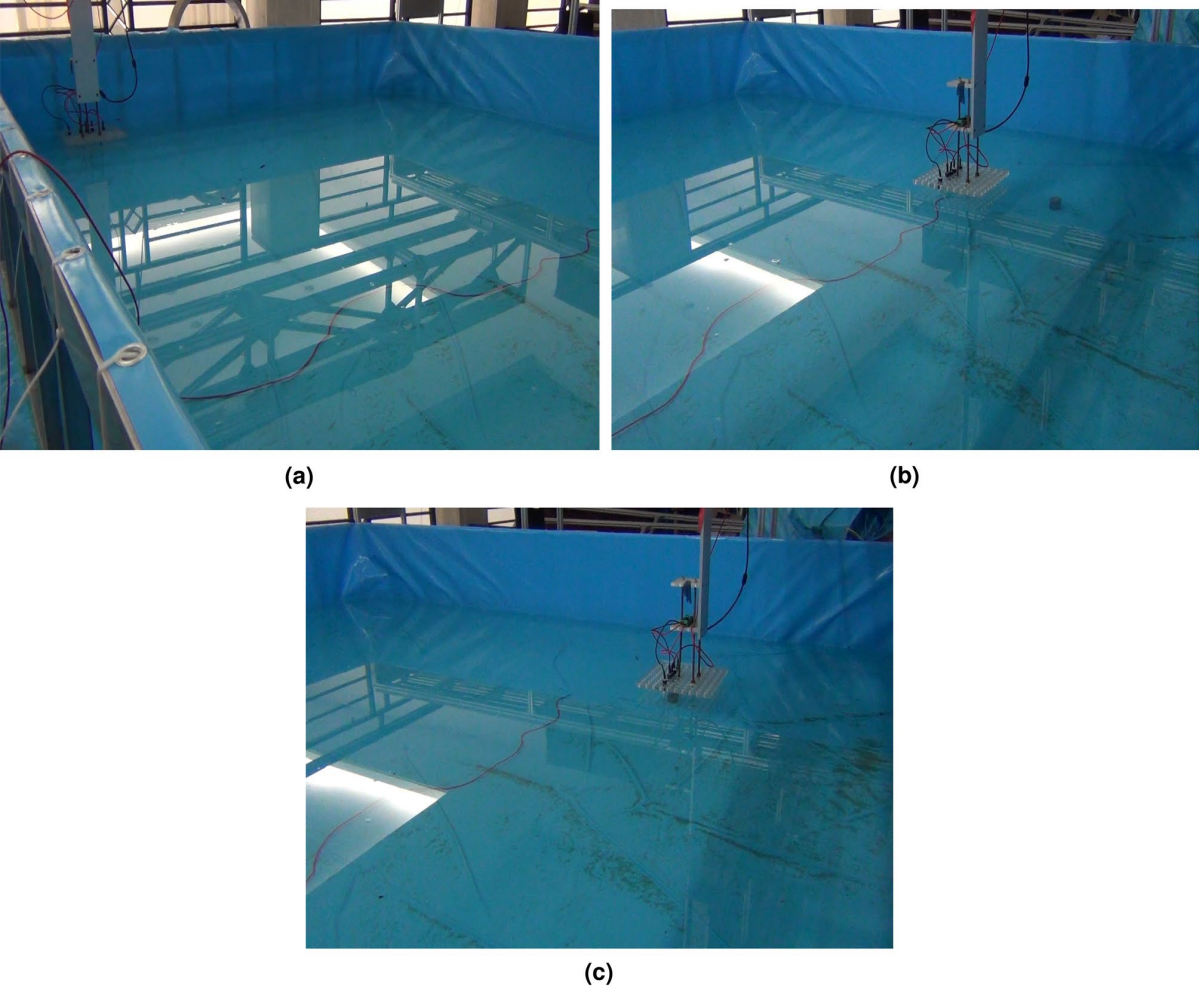
Screenshot of part of the joint active–passive electrolocation experiment. (**a**) Positioning device starting position; (**b**) reaching the signal source position; (**c**) targeting success.

In the experiments of joint active–passive electrolocation algorithm, the hill climbing algorithm is firstly used to search the signal source location to make the localization device move near the target, and then switch to the PSOSA combined optimization algorithm to make the localization device move to the target location. A typical localization path in the experiment is shown in Fig. 11. The red dots indicate the position after each iteration update of the hill-climbing algorithm, the blue dots are the position after the iterative update of the PSOSA combined optimization algorithm, and the grey arcs are the equidistant lines centered on the target object. Figure 11a shows the joint localization path, and Fig. 11b shows a zoomed-in view of the localization path in the active electrolocation part of Fig. 11a. With the help of the hill climbing algorithm, the localization device started searching from the corner of the pool and gradually approached the target object, and after 4 iterations, the localization device successfully approached the vicinity of the target. Then the PSOSA combined optimization algorithm came into play, and through 12 iterations, the positioning device finally reached the target location and achieved the precise positioning of the target. The images taken from the positioning experiment verification video are shown in Fig. 12. Where FigurFig. 12a shows the moment of electrolocation onset, Fig. 12b the moment of successful passive electrolocation guidance, and Fig. 12c the final position of active electrolocation. After measurement, the positioning error was within 0.05 m, indicating that the positioning method has high accuracy and reliability.

## Discussion

The above three experiments verified the feasibility of the three algorithms designed for underwater localization. The results show that all three algorithms can successfully localize the target object in no more than 16 iterations, which proves that they have faster localization speed. Among them, the number of iterations of the joint active–passive electrolocation algorithm is higher than that of the independent hill-climbing algorithm and the PSOSA combined optimization algorithm. This is because the joint active–passive electrolocation algorithm, which is a combination of the latter two algorithms, was experimented in a larger pool, and a larger experimental range may increase the number of iterations. However, compared to the other algorithms listed in Table 1, the average number of iterations of the joint active–passive electrolocation algorithm is still relatively low, although it has a larger localization range, which indicates the fast localization speed of the proposed method.

During the experimental localization process, the position of the passive electric field switching to the active electric field is controlled by human. Under our research conditions, as shown by the simulation results in Table 2 and the experimental results in Fig. 8, the PSOSA combined optimization algorithm can successfully locate the target object with fewer iterations when the detection device is within 50 cm from the target object. Therefore, for the convenience of the experiment, in the experiment of the joint active–passive electrolocation algorithm, it is set that the passive electrolocation switches the active electrolocation when the detection device is within 50 cm from the target object. In fact, it is also possible to use the PSOSA combined optimization algorithm to locate the target object beyond the 50 cm range, but due to the distance limitation of the active electric field, the distance is too large to increase the number of iterations of the localization algorithm and reduce the success rate of the localization, because this is not the focus of the discussion in this paper, we will not go into detail here, interested readers can further study this.

**Figure 13:**
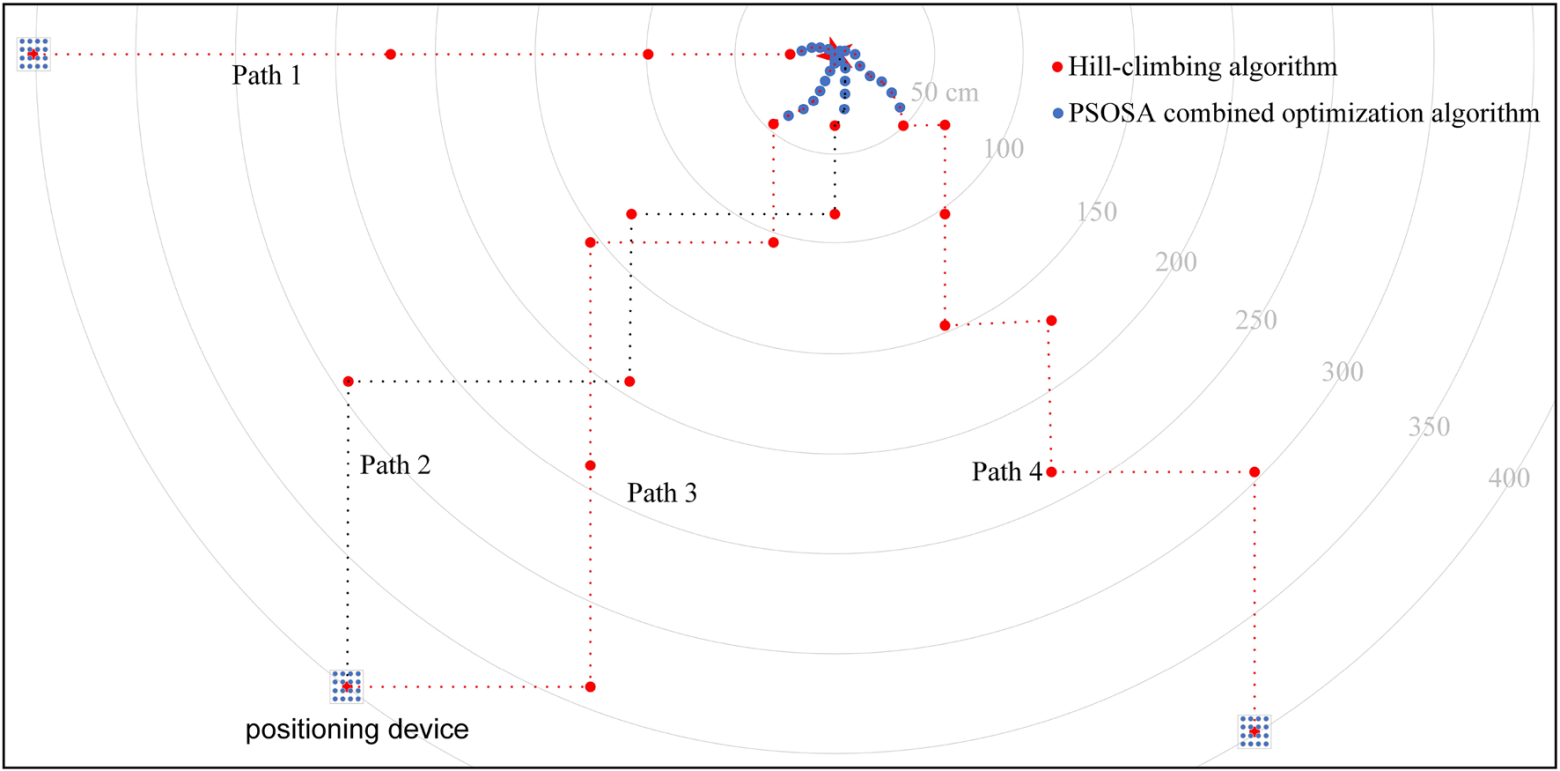
Schematic diagram of the joint active–passive electrolocation algorithm for multiple localizations.

We only demonstrated the results of the algorithm in a pool of size 4 m $\times$ 2.9 m $\times$ 0.5 m and only one localization path was shown. However, due to the wide range of passive electric field detection, the actual joint active–passive electric localization has a larger localization range than that in the experiment. In actual localization, the randomness of the particle distribution during initialization makes the detection device localize the same target object at the same location several times, with different localization paths each time, and the number of iterations will also change. In order to facilitate the understanding of the positioning process of the joint active–passive electrolocation algorithm, we draw a multiple positioning schematic as in Fig. 13, in which the starting point of the four localizations is 4 m from the target object. localization path 1 indicates that after four iterations of the hill-climbing algorithm, the detector device has been moved to the object under test at a distance of less than 50 cm, and then switched to the PSOSA combined optimization algorithm for active electrolocation; localization path 4 indicates that after six iterations of the hill-climbing algorithm, the detection device is just 50 cm away from the measured object, and then switches to the PSOSA combined optimization algorithm, which successfully locates the target object after a different number of iterations from Path 1; whereas Localization Paths 2 and 3 are the two localization processes of the detection device at the same location, which indicates that the localization paths of the detection device are not the same even when the detection device is at the same location.

Although this paper only discusses the localization of an aluminum cylinder as the target object in fresh water, and due to the different conductivity of water, the response of the same object to the electric field is different^35,36^. However, according to the mechanism of the algorithm, even in seawater, a charged object in water can still be successfully localized using the hill-climbing algorithm; the PSOSA combined optimization algorithm can still successfully localize an object with similar characteristics to the response of an aluminum cylinder to an active electric field in freshwater (e.g., Fig. 2b). Since the active–passive switching can be controlled artificially, this method can be applied to the following three cases: (1) when the target object is a charged body and the response characteristics to the active electric field are not similar to those in Fig. 2b, the hill-climbing algorithm can be used to localize it. (2) When the target object is not charged and the response characteristics to the active electric field are similar to Fig. 2b, it is directly switched to PSOSA combined optimization algorithm to achieve proximity localization. (3) When the target object is electrically charged and the response characteristics to the active electric field are similar to that of Fig. 2b, the joint active–passive electrolocation algorithm can be used to achieve long-distance localization.

The method proposed in this paper has higher localization accuracy than acoustic methods, farther localization distance than optical methods, and simple algorithm with low cost. Combined with the fact that the active electric field has the ability to recognize the material, size and shape of the object under test, this method has great research value in locating an unknown discharged object at a long distance and with high accuracy and recognizing the characteristics of the object. For example, this method is applied to underwater work units for salvaging important objects such as flight data recorder. When the distance is far, passive electrolocation is used to search for the approximate position of the target object (the source of the electric field signal is not necessarily on the target object due to the possible disintegration of the object), so that the underwater robotic working unit moves to the vicinity of the target object, and then switches to active electrolocation, so that the underwater robotic working unit can accurately locate the target and then recover it.

In summary, the algorithm designed in this paper is an effective underwater electrolocation algorithm.

## Conclusions

In the present study, we design a novel joint active–passive electrolocation bionic algorithm that can be used for underwater high-precision and long-range positioning based on the characteristics of active electrolocation with high positioning accuracy and passive electrolocation with long positioning distance. One of the passive electrolocation component employs a hill climbing algorithm to facilitate long-range localization, whereas the active electrolocation element utilizes a particle swarm optimization simulated annealing (PSOSA) combined optimization algorithm for proximity positioning in order to improve the positioning accuracy. Furthermore, the PSOSA combined optimization algorithm, an amalgamation of the particle swarm optimization algorithm and the annealing algorithm, demonstrates a high localization success rate and reduced average iteration count. The practicability of the hill climbing algorithm, the PSOSA combined optimization algorithm, and the joint active–passive electrolocation algorithm is corroborated through a series of experiments. The empirical findings indicate that the suggested algorithms yield considerable localization range and accuracy, rendering them suitable for applications in underwater robotics, search and rescue operations, and naval military endeavors. Nevertheless, the localization velocity of the proposed algorithm is inferior to that of the hill climbing algorithm and the PSOSA combined optimization algorithm individually. Future research will be conducted to examine the localization speed in greater depth.

### Consent to participate

All authors agree to publication.

## Data Availability

The authors declare that all data supporting the findings of this study are available within the article.
